# Traumatic Brain Injury: At the Crossroads of Neuropathology and Common Metabolic Endocrinopathies

**DOI:** 10.3390/jcm7030059

**Published:** 2018-03-14

**Authors:** Melanie Li, Swetlana Sirko

**Affiliations:** 1 Physiological Genomics, Biomedical Center (BMC), Institute of Physiology, Medical Faculty of the Ludwig-Maximilian University Munich, 82152 Planegg-Martinsried, Germany; Melanie.Li@med.uni-muenchen.de; 2 Institute of Stem Cell Research, Helmholtz Center Munich, Research Center for Environmental Health GmbH, 85764 Neuherberg, Germany

**Keywords:** neuroendocrinology, endocrine disorders, brain injury, critical illness, diabetes mellitus, obesity, metabolic syndrome, hypothyroidism, hypothalamic-pituitary axis

## Abstract

Building on the seminal work by Geoffrey Harris in the 1970s, the neuroendocrinology field, having undergone spectacular growth, has endeavored to understand the mechanisms of hormonal connectivity between the brain and the rest of the body. Given the fundamental role of the brain in the orchestration of endocrine processes through interactions among neurohormones, it is thus not surprising that the structural and/or functional alterations following traumatic brain injury (TBI) can lead to endocrine changes affecting the whole organism. Taking into account that systemic hormones also act on the brain, modifying its structure and biochemistry, and can acutely and chronically affect several neurophysiological endpoints, the question is to what extent preexisting endocrine dysfunction may set the stage for an adverse outcome after TBI. In this review, we provide an overview of some aspects of three common metabolic endocrinopathies, e.g., diabetes mellitus, obesity, and thyroid dysfunction, and how these could be triggered by TBI. In addition, we discuss how the complex endocrine networks are woven into the responses to sudden changes after TBI, as well as some of the potential mechanisms that, separately or synergistically, can influence outcomes after TBI.

## 1. Introduction

The concept of active participation of the central nervous system (CNS) in hormonal communication throughout the body is closely connected to the historic development of endocrinology. The assumption that the brain works in tandem with the endocrine system to maintain the balance of various systems in mammals dates back to the second to eighteenth centuries AD [1,2]. However, active participation of specialized areas of the brain in the integration of endocrine, autonomic, and behavioral responses was demonstrated by Geoffrey Harris in 1970. Harris was the first to discover the communication between the hypothalamus and the pituitary gland [3,4], establishing the idea of a hormonal axis between the CNS and the rest of the body. This finding opened many doors to exploring the influence of neural activity on endocrine secretion, as well as discovering how alterations in neuroendocrine functions can actually have an impact on fundamental physiological processes within the body, including homeostatic balance, growth, reproduction, energetics, and metabolism. Besides the hypothalamus, the pituitary and pineal glands provide key communication and control links between the two main systems, the nervous and endocrine systems, and have been classed as the primary elements of neuroendocrine integration. The human pituitary gland is a pea-sized appendix of the diencephalon lying at the base of the brain. It consists of two parts, the adenohypophysis and the neurohypophysis. The latter directly secretes potent neuropeptides into the circulatory system after they are transported axonally from hypothalamus nuclei, where the neurosecretory cells reside. The adenohypophysis receives releasing or inhibiting factors from other hypothalamic nuclei via a vascular portal system, which themselves regulate the secretion of a number of hormones into the blood, covering a broad spectrum of defined functions [5]. It is thus obvious that damage to this small hormone gland can cause long-lasting and even permanent consequences for the whole body.

Taking into account that signs of neuroendocrine dysfunction, such as low blood pressure, reduced heart rate, anemia, constipation, cold intolerance, loss of muscle mass, depression, and poor memory, are not uncommon in the context of traumatic lesions to the brain [6], and due to the fact that the rates of pituitary dysfunction among survivors of traumatic brain injury (TBI) are approximately 37–59% [7,8], the acute or chronic (neuro) endocrine dysfunctions induced by TBI present a range of consequences that should not be neglected. As the mean annual incidence rate of hospitalized survivors after TBI in industrialized European countries is about 262 per 100,000 persons per year [9], the pressing need to advance research in (neuro) endocrine aspects is thus more than obvious. This is especially important when considering the prediction of the World Health Organization (WHO) that TBI will become the third leading cause of death and disability in the world by 2020 [10]. Although enormous progress was made during the past decades, TBI still represents a significant medical problem. It remains a challenging era for the clinical development of improved combinatorial therapeutic strategies for TBI patients, since a number of pharmacotherapeutic approaches designed to modulate single groups of mechanisms have failed in clinical trials, despite showing preclinical promise [11]. Rationales for combining treatment strategies result from the notorious complexity and heterogeneity of disease processes of tissue injury itself. Systemic and extraneuronal effects of trauma provide an additional rationale, given that these are often the actual causes of death in brain-injured patients [12]. In general, the pathophysiological mechanisms that occur in the post-traumatic brain can be subdivided into primary and secondary damage cascades. Each cascade involves a different set of processes, which often overlap. The primary injury occurs during the initial phase and is refractory to most treatment, whereas the multivariable secondary cascade, coming along with TBI-induced neuronal apoptosis, necrosis, neuroinflammation, and massive gliosis [13], is more treatable and, at least in part, preventable [14]. That is why recent TBI research is focusing on improving therapeutic guidelines in order to reduce inflammation and neuronal loss, as well as enhancing neuronal survival and promoting neuroprotective and neurogenic capacities [15,16].

Given that the prognosis after TBI is strongly dependent on both the anatomical location and the severity of damage, assessing these two parameters is fundamental to clinical management and the design of treatment trials, saving patients from unnecessary, often harsh suffering. To date, the “key points” used to classify the severity of TBI include assessment of consciousness level by the Glasgow Coma Scale (GCS) [17], assessment of structural damage revealed on neuroimaging scans (CT classification) [18], and different clinical biomarkers, mainly for cerebrospinal fluid (CSF) or blood-based assays [19]. All of these biomarkers aim to evaluate TBI and accelerate the diagnostic procedure, as they allow proof and follow-up of the degree of tissue damage based on the level of axonal injury (e.g., Tau or NFL, the light subunit of neurofilament protein) [20,21], neuronal loss (e.g., neuron specific enolase (NSE) or γ-enolase) [22], damage of astroglial cells (S100 calcium-binding protein B (S100β) or glial fibrillary acidic protein (GFAP), both astrocyte-specific proteins) [21,23], neuronal damage (e.g., UCH-L1, ubiquitin C-terminal hydrolase L1 involved in the removal of proteins in pathological conditions in neurons) [24], and synaptic dysfunction (SBDPs, the synaptotagmin breakdown products released during synaptic dysfunction) [25,26]. The expression kinetics and patterns of these biomarkers appear to be a good index of the extent of injury; however, none of them reliably mirror the consequences to the organism as a whole [25,27]. Given the magnitude of the post-traumatic structural, chemical, and metabolic changes that may be intimately related to a variety of adverse endocrine conditions (preexisting or induced by acute brain injury), which may further contribute to the development of systemic insufficiency, there is a great need for reliable diagnostic and prognostic evaluation of hormonal parameters after brain trauma, especially in patients with moderate to severe TBI. This is not only reasonable, but essential, given the role of endocrine processes in systemic manifestations of brain injury [6,28] and the fact that the treatment of severe TBI cases is mainly reduced to focal neurological problems, focusing on avoiding secondary insults and improving cerebral blood flow and metabolism [16]. For all these reasons, this review approaches a number of ‘endocrine’ aspects of brain injury, as they appear to be involved in many, if not most, of the substantial problems leading to elevated risk for poor outcome in TBI.

## 2. All Ranges of TBI Can Cause Significant Hormonal Dysfunction

During the last 15 years, most of the retrospective and prospective studies on TBI revealed an increased prevalence of post-traumatic neuroendocrine dysfunctions that can produce lifelong deficits to varying degrees. Pituitary dysfunction following traumatic brain injury has recently received considerable attention, partly because approximately 50% of TBI patients may develop pituitary deficiencies [29]. However, the use of systemic pituitary hormonal monitoring has been taken up slowly [30,31], and it is still not part of standard screening or outcome prediction protocols after TBI [32].

Hypopituitarism (HP) is defined as a neuroendocrine failure that may involve the loss of one, several, or all of the pituitary hormones due to hypothalamic-pituitary lesions, regardless of its origin. Based on the degree and severity of hormone deficiency, HP can be subdivided into partial and complete (also known as panhypopituitarism) forms [33]. In general, HP is a relatively rare condition, affecting 46 per 100,000 patients [34], and is mostly caused by adenomas or other tumorous lesions associated with higher mortality [35]. It should also be taken into account that cranial or total-body irradiation contributes to a higher risk of developing progressive and irreversible HP [36]. Of particular interest is a causative relationship between TBI and HP, which was suggested in 1918 by Paul Cyran [37]. Although until recently there has been relatively little data on the prevalence of HP, according to the current study more than half of all investigated TBI patients developed hormonal abnormalities starting three months after TBI [29], and up to 50% of them were indeed diagnosed with HP. Also, the fact that almost one-third of patients, who died very shortly after the brain trauma, showed pituitary gland infarction [38] clearly demonstrates that pituitary insufficiency after TBI is much more frequent than previously considered [39]. Even when the risk of developing HP increases proportionally to the severity of TBI [40,41], the pooled prevalence of post-traumatic HP is still estimated to be ~17% in mild, ~11% in moderate, and ~35% in severe TBI cases [39]. Taking into account that approximately 80% of all head injury cases are categorized as mild TBI [42,43], this form of TBI carries a substantial risk of developing post-traumatic HP [7,44,45].

In clinical practice, mild TBI in patients with a condition after head trauma has been defined by a GCS score of 13–15 [17], and more recently by the appearance one of the following symptoms: loss of consciousness for less than 30 min, loss of memory of events immediately before or after the trauma, or impairment of the mental state for up to 24 h [46]. In these cases, patients are often discharged very early or do not seek medical help at all, because the symptoms are very unspecific or transient. Although most clinical studies focus only on the impact of moderate to severe TBI on long-term pituitary dysfunction, a few groups are investigating this underestimated clinical picture. Here, studies have shown much higher HP prevalence after TBI than one would assume. Indeed, data from Tanriverdi and colleagues indicated that the percentage of patients developing hormonal dysfunction after repetitive sports-related to mild TBI ranged from 22.1% [47] to 51.4% [8] in total, and even a reduction in the volume of the pituitary gland could be observed [48]. Furthermore, a study from Kelestimur et al. reported growth hormone (GH) deficiency in 45% of boxing athletes [49], and Ives et al. found several hormonal axes affected in a young patient after multiple soccer-related concussions [50]. These dimensions strongly suggest the need to standardize screening protocols, on which more work has been done lately [51].

The manifestations of post-traumatic HP vary according to not only the severity of the trauma, but also the time elapsed since the trauma [40]. Because partial or complete hypopituitarism may occur during acute phases, but also months to years after TBI, an endocrine assessment for pituitary function in the acute phase as well as prospective evaluations 12 months after the traumatic event are recommended for all TBI patients regardless of severity [30,31]. Since the screening of pituitary functions poses diagnostic challenges, in many cases post-traumatic HP still remains an often overlooked problem [40,41]. Therefore, understanding the pathophysiological processes underlying the development of dynamic characteristics of HP is critically important. Aside from the injury-induced focal or systemic inflammatory responses inducing degenerative processes, other factors may contribute to a gradual evolution of dysfunction [41]. Several previous studies evaluated an apparently greater resistance of the adrenal, thyroid, and posterior neurohypophyseal axes, while the gonadotrophic and somatotrophic axes are more frequently affected, with the latter seeming to have a more chronic character [29,52,53,54]. Still, identifying the key molecular and pathophysiological mechanisms regulating the development of HP is necessary to establish evidence-based diagnostic strategies to prevent a relatively straightforward adoption of hormone deficiency after brain injury. This goes especially for patients with the most predictive pathogenic factors for the development of HP, such as basilar skull fracture or diffuse axonal injury, increased intracranial pressure, and diffuse brain swelling, as well as an (evacuated) intracerebral hematoma [7,53,55,56,57]. It is worth noting here that many of these symptoms might primarily result from the brain injury itself, and grave structural lesions causing impaired vigilance or disorientation could mask early HP signs. In addition, it is important to remember that manifestations of hypopituitarism, in particular with isolated hormone deficiencies as in TBI, could be mild or subtle. Thus, it is likely that most HP patients remain undiagnosed and therefore untreated [39].

Clinical suspicion suggesting screening of TBI patients and knowledge of risk factors, therefore, plays a critical role in the diagnosis of post-traumatic hypopituitarism. The effort to understand TBI-related hormonal dysfunctions and the available strategies to treat the consequences should also be based on precise research of long-term effects. It is noted in this regard that a major percentage of TBI patients do show long-term memory and concentration deficits, depression, fatigue, and loss of emotional well-being comparable to symptoms in endocrine disorders such as GH deficiency, hypogonadism, adrenal insufficiency, and hypothyroidism [58,59,60]. A correlation is very probable, as HP also leads to lethargy, severe fatigue, and other neuropsychiatric manifestations. GH deficiency as well as sex-steroid deficiency can weaken the patient, whereas glucocorticoid deficiency can be life-threatening [38].

## 3. Systemic Endocrine Disorders Can Impact the Outcome of TBI

**Figure 1:**
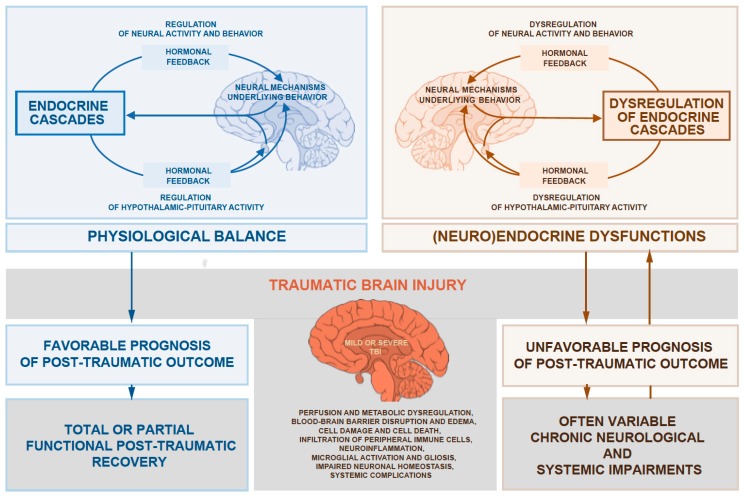
The relations schematic shows the concept of a cause-and-effect relationship between the outcome of traumatic brain injury (TBI) and a preexisting (neuro) endocrine state that appears to be involved in many, if not most, of the substantial problems leading to elevated risk for poor outcome in traumatic brain injury.

Over the last decades, a growing number of studies have shown substantial evidence for endocrine dysregulation due to injury-induced hypothalamic-pituitary disturbances. However, there are still many important aspects that are not fully understood, when taking into account that multiple risk factors, including preexisting common metabolic endocrinopathies, are involved in the post-traumatic outcome (Figure 1).

### 3.1. Glucose Metabolism

At the pathophysiological level, TBI induces massive changes in glucose metabolism, associated with a decrease of oxidative metabolism due to severely impaired mitochondrial function [61,62]. At the same time, anaerobic glycolysis, the main energy source within the injured brain parenchyma, is not depressed for quite a while after the laceration, thus resulting in ‘relative hyperglycolysis’, a state with increased levels of pyruvate and lactate [63]. As a consequence, energy-generation efficiency decreases significantly, since one molecule of glucose is converted to only a fraction of energy-storing ATP molecules when compared to oxidative processes [64]. Thus, it is obvious that even small fluctuations of intracellular glucose in the cells can quickly lead to an imbalance regarding energy demand.

#### 3.1.1. How Insulin Affects the Brain

Insulin initiates cellular glucose uptake by binding to membrane receptors, resulting in their phosphorylation and subsequent recruitment of substrates binding phosphoinositid-3-kinase (PIP3). The latter then activates a variety of other kinases, inducing the translocation of glucose transporters, such as GLUT4, to the cellular membrane, among other actions. Only in the past few years has the widely accepted view of the brain as an insulin-independent organ been challenged [65,66]. Studies have shown not only functional influence [67,68,69,70,71], but a significant induction of glucose uptake, especially in the telencephalon [65]. Insulin indeed can modify brain cells, either directly via specific receptors [72], by passing through astrocytes lining the Virchow–Robin space or tanycytes in the walls of the ventricles into the cerebrospinal fluid [66,73,74], or via the median eminence [74]. In the CNS, glucose transporters can be found widely in glial cells [66], which are necessary for a functioning nervous system, especially when it comes to metabolism, neural repair, immune defense, functionality of the blood-brain barrier (BBB), and regulation of neuronal activity. In particular, parenchymal astrocytes surrounding capillaries respond to changes in blood glucose levels by up- or downregulation of GLUT1 transporters [75,76]. To what extent the insulin-dependent uptake is accomplished by different cell types throughout the brain, however, remains unclear.

#### 3.1.2. Hyperglycemia as a Predictor of Mortality Following TBI

The fulminating inflammatory state following TBI has systemic but also specific effects on the CNS. It is especially intriguing that, among other aspects of the evolution of traumatic injury, inflammatory cytokines may affect insulin-signaling pathways, potentially disrupting glucose availability after TBI [77]. Tumor necrosis factor-α (TNF-α), for example, is potent for almost half of insulin-induced phosphorylation, impairing the display of transporters on the cell surface so that cells can hardly meet their glucose demands [78,79]. Additionally, the levels of GH (a hormone that acts very similarly to insulin) elevate during the acute phase after severe injury, and concomitantly induce a state of peripheral resistance. This constellation is potent in enhancing an insulin-antagonizing effect, leading to even more elevated glucose levels [80]. Hyperglycemia, defined as excessively increased levels of blood glucose, is not only a marker of tissue damage, but also a reliable and independent predictor of mortality in TBI patients [81,82,83,84]. This at first seems to be a paradox, since the circulatory system embodies the vehicle to transport nutrients to the brain, but excessively high glucose levels only occur when cells are unable to ensure proper uptake. This dysfunction is especially associated with pathologic insulin deficiency or resistance, as is the case in patients with diabetes mellitus (DM) [85]. Also, in the acute phase of any critical illness, so-called stress diabetes may develop, enhancing the extent of hyperglycemia: after a dramatic acceleration of hepatic glucose production to meet the organism’s energy demands, hyperinsulinemia develops, but is unable to maintain stable glucose levels, resulting in insulin resistance due to reasons mentioned below. In the immobilized patient, activity-stimulated glucose uptake in skeletal muscles nearly disappears, a feature that strongly contributes to the state of critical illness [80].

More obviously, hyperglycemia is harmful, as it can compromise microcirculatory blood flow and lead to abnormally high BBB permeability. It promotes inflammation and immunosuppression, and triggers volumetric balancing issues such as hypovolemia [86]. However, scientists and doctors seem divided over glucose control in patients with severe trauma. There are two treatment paradigms for critically ill patients and those with severe TBI: conventional glycemic therapy (CGT) (maintenance of blood glucose < 180 mg/dL using subcutaneous insulin, and if the level exceeds 220 mg/dL, intravenous insulin is added) and intensive insulin therapy (IIT) (strict maintenance of blood glucose between 80 and 110 mg/dL) [87]. IIT has been shown to reduce mortality in critically ill patients [85] and also the risk of developing a severe complication called critical illness polyneuropathy (CIPNP), depress intracranial pressure, suppress seizures, and can, all in all, improve long-term prognosis after hospital discharge [88]. Also, a reduction of mortality by almost one-third [89], fewer infectious complications, and shorter intensive care unit (ICU) stays have been observed [90]. However, many research groups have found no difference in outcome between the two paradigms, but instead dangerous hypoglycemic episodes were noted in IIT treated patients, especially in injured regions [91], which can be fatal as well [92,93,94,95,96]. A large international study by the Normoglycemia in Intensive Care Evaluation-Survival Using Glucose Algorithm Regulation (NICE-SUGAR) committee showed higher mortality, mostly due to cardiovascular complications, when compared to CGT controls [87]. Although these studies cannot be compared directly, since there are large differences in terms of cohort size, treatment paradigms, treatment intensity (i.e., ICU vs. regular unit), and preexisting patient conditions, concurrent glucose infusions might be needed to protect patients from severe hypoglycemic episodes [77], along with richer enteral nutrition [93]. Individualized blood glucose control guided by microdialysis monitoring of brain glucose levels could be an alternative [32], but this requires further study and establishment of protocols.

#### 3.1.3. Diabetes Mellitus as a Risk Factor for Higher TBI Mortality

Nowadays, almost every tenth person in the adult population is diagnosed with diabetes mellitus (DM). In 2012, DM caused approximately 3.7 million deaths, almost half of them occurring in patients younger than 70 years of age. The wide range of complications, including heart attack, stroke, kidney failure, leg amputation, and vision damage, generate huge costs for most countries’ healthcare systems [97]. Referring to the paragraph above, it is obvious that insulin-related pathologies have an impact on the brain, regardless of whether the clinical picture is characterized by insufficient insulin production due to autoimmune processes (type I DM), a state of absolute deficiency, or by resistance to actions of the secreted hormone (type II DM), a state of relative deficiency.

Several experimental approaches were established to explore this hypothesis using animal models. However, the relationship between diabetes and neuropathy is still far from observable, partly due to the lack of suitable animal models that mimic human etiology and pathogenesis. One possible mechanism by which nutritional characteristics or habits interact with damage and the brain was suggested by Hoane et al. This study revealed that a high-fat sucrose diet in animal models had a significantly negative impact on the outcome of brain injuries in terms of lesion size and neuronal and behavioral plasticity, probably due to a reduction of brain-derived neurotrophic factor (BDNF) [98]. BDNF is the most ubiquitous member of the family of neurotrophins in the CNS, which trigger phosphorylation cascades that promote protein synthesis, axonal growth, dendritic maturation, synaptic plasticity, and neuroprotection [99]. It is thus not surprising that the detected loss of hippocampal neurons due to increased apoptosis could gravely impair cognitive ability and performance, and even cause a loss of brain weight [100]. On the other hand, such extent of neurodegeneration in diabetic mice could also be the consequence of imbalanced glial activation throughout different brain regions, creating massive neuroinflammation and, in the worst cases, a neuron-threatening environment [101,102]. Moreover, DM can potently cause dysregulated glucose utilization in astrocytes [103], ultimately leading to an energy-deprived state in which the enrichment of reactive oxygen species (ROS) results in damage of neuronal DNA, leading to neuronal death [104]. The aberrant accumulation of ROS is also related to the infiltration of neutrophils, which induces an inflammatory response that, in turn, increases the generation of ROS, initiating the oxidative stress cascade and, consequently, large neuronal cell loss [105].

Furthermore, consonant with the clinical picture, enrichment of peripheral circulating ketone bodies, which can be taken up by astrocytes as an alternative energy fuel [106], may also trigger specific aspects of reactive astrogliosis and the formation of gliotic foci within the brain parenchyma [107]. In support of this, reactive states of glial cells in diabetic animals were paralleled with significant increases in the number of astrocytes and microglia throughout different hippocampal regions [100]. Since astrocytes serve as biosensors for almost any type of neuropathology, and reactive astrogliosis is the prototypical response of CNS to diverse types of injury mediated by various cell types and involves the activation of microglia [108,109], these findings could be linked to considerable neuronal damage within the different brain regions that go hand-in-hand with behavioral impairments in DM-affected animals. Although the data obtained from animal studies cannot be directly transferred to humans, they do contribute to a better understanding of pathophysiological mechanisms and provide prime candidates to foster the development of new pharmacological drugs.

While we focused on experimental DM paradigms above, it is important to mention that the gathered data strongly suggest a negative effect of a diabetic state not only on the capacity of neuronal repair after TBI, but also on the preexisting resilience against neuronal trauma of affected patients. In line with this, TBI patients with DM had significantly higher mortality and longer hospital stays than nondiabetic TBI patients. Furthermore, there is strong evidence suggesting a correlation between low insulin levels and lethality. This also appears to be independent of related comorbidities, since individuals with type I DM showed higher mortality than those with type II DM [77]. Considering the growing prevalence of DM, the emerging clinical relevance is not hard to see.

It is thus obvious that a first clinical approach could be even closer monitoring of cerebral gliosis, either by using biological markers or via imaging, combined with an intensive anti-inflammatory treatment plan that is escalated as needed. Of particular importance is not only the control of blood glucose levels, but also the maintenance of physiological insulin levels in TBI patients presenting with deficiencies of the latter. Still, further effort is required to better reconstruct pathophysiological changes that corroborate the level of impact on TBI, so that therapeutic guidelines can be precisely improved.

### 3.2. Obesity

The WHO states that more than 650 million people in the world were obese in 2016, a number that continues to increase year by year, conspicuously comprising children and adolescents [110]. A major study analyzing health trends in the US population between 1999 and 2008 estimated that more than one-third were defined as obese, i.e., having a body mass index (BMI) greater than 30 kg/m^2^ [111]. These numbers, and especially the strong involvement of younger generations, dramatically show the emerging relevance of treating affected patients, since obesity is associated with a wide range of health concerns [112].

#### 3.2.1. Obesity Causes Chronic Low-Grade Systemic Immunological Dysfunction

Immunologically, the long-standing idea of chronic low-grade inflammation in obese patients has gained wide acceptance [79,112,113,114,115]. Elevated concentrations of the pro-inflammatory agents TNF-α, interleukin (IL)-6, and IL-8 can be found, presumably arising from adipose tissue [112,116,117]. In addition, serum levels of anti-inflammatory signaling molecules, such as IL-10 and adiponectin, seem to negatively correlate with the level of adiposity [118]. Similar effects can be found at the cellular level, with more macrophages accumulating in atherosclerotic plaques when compared to individuals with normal BMI [112]. Such features probably lead to a higher risk of developing cardiovascular diseases [119] and a greater prevalence of pulmonary complications, associated with a more active state of pulmonary alveolar macrophages [120]. At the same time, studies have shown worse long-term adaptation to pathogens, along with a reduction in the number of CD8+ T cells, which are on top mostly dysfunctional [112]. This could explain the imbalance of cytokine levels in general. Also fibrinogen, an important part of the organism’s acute response, may play a prominent role in adiposity-associated hemostatic imbalance [113], leading to hemodynamic complications and disturbances such as thrombosis or embolism [121,122].

#### 3.2.2. Obesity Can Cause Hypothalamic and Diffuse Brain Inflammation

In the murine CNS, the hypothalamus accumulates triacylglycerols, diacylglycerols, and ceramides after high-fat diet (HFD) feeding [123], causing localized inflammatory processes that have been observed in obese individuals [124,125,126,127]. Short-term HFD is capable of quickly inducing hypothalamic insulin resistance [125], whereas long-term HFD was shown to activate inflammatory signaling pathways, including transcriptional mediators such as c-Jun N-terminal Kinase (JNK) and nuclear factor-κβ (NF-κβ). When activated, both of these factors are generally assumed to promote the production of pro-inflammatory cytokines [128,129,130], impairing the insulin signaling pathway [77,79]. Together with widely upregulated levels of IL-β and CCR2 within the brain parenchyma [131], all these factors evoke a damaging environment that may contribute to the induction of gliosis, which many studies have now focused on.

A study by Gao and colleagues has investigated the effects on the hypothalamic microglia of HFD-fed mice [132]. Compared to control animals, a significantly increased number of Iba1+ microglial cells were detected, which tend to switch toward an activated phenotype and co-express a marker associated with phagocytosis (e.g., CD68). Interestingly, treatment of primary cultured hypothalamic microglia obtained from control animals with serum extracted from HFD-fed mice induced gene expression of TNF-α and IL-1β. These processes tend to correlate positively with cytokine concentrations, as genetically modified animals with higher release rates show even stronger reactions in situ. Similar reactions were also observed by Gupta et al. in cultured astrocytes [133]: the release of cytokines could also be induced upon exposure to saturated long-chain fatty acids, acting as an alternative fuel, but disturbed the metabolic lipid balance [106]. On top of that, exaggerating IL-6 levels can disturb normal metabolism in parenchymal astrocytes, gravely affecting their functioning [134].

A further and particularly alarming finding reported in a genetic murine obesity model is leakage of the BBB, likely triggered by gliosis [135]. These studies raise the tantalizing possibility that a subset of leukocytes derived from a myeloid progeny [136] are capable of entering the brain parenchyma [137], potentially reinforcing the already excessive inflammatory cascade. Given that oxidative environments, such as an inflammatory milieu, can precondition the brain for neurodegeneration [102], it is not surprising that hypothalamic neurons are strongly influenced by such conditions. In line with this, an ensheathment of synapses and reorganization of synaptic input and a modification of neurotransmitters are related to diet-induced hypothalamic reactive gliosis and obesity [138,139]. Especially striking are the loss of resident neurons and impairment of local neuronal plasticity due to apoptosis of neural stem cells within the adult neurogenic niches [140]. Similar trends have been detected in the course of early hippocampal neurogenesis, possibly causing cognitive and learning impairments [141], which is particularly intriguing, as the latter is a region very distant from the hypothalamus. Given the above considerations, it clear that obesity, classically associated with the disruption of pathways controlling lipid and glucose metabolism, also embodies an inflammatory condition that results from a complex sequence of events and is, in large part, responsible for the development of secondary brain damage. One group has investigated the impact of HFD-induced obesity on the vulnerability of the post-traumatic murine brain in regard to secondary brain damage [142]. Serum cortisol levels in obese male mice were almost halved 30 days after a mild concussive event; however, in control animals, secretion rates increased by 26%. This is compatible with a nonphysiological stress reaction of the organism in obese mice, maybe due to a damaged adrenal axis. Furthermore, obesity-induced massive microgliosis could, again, be found not only in the hypothalamus, but also throughout distant areas of the brain, i.e., the cerebral cortex and the corpus callosum. Such results indicate that the manifestation of diffuse neuroinflammation is a likely cause of behavioral impairment. These data strongly suggest the potential gravity of obesity-induced CNS vulnerability, which can have devastating, long-lasting, and even permanent consequences in obese TBI patients. This is in accordance with the observations in two recently published studies demonstrating that mortality and post-traumatic complications were common in patients predisposed to obesity vs. non-obese trauma patients, despite having longer hospital and ICU stays [143,144].

#### 3.2.3. Consequences of Obesity on TBI Outcomes

Although the detrimental effects of obesity on overall health are well understood [145], exactly how obesity affects TBI outcomes is not. Despite an increased relative risk of fatal outcomes in obese patients after frontal car crashes [146], there is also evidence showing that the impact of obesity on the outcome of TBI is not nearly as grave as one would assume, even though it does exist [147]. A study by Arbabi et al. examined outcomes for adult and pediatric blunt trauma patients [148]. Here, they found obesity to be an independent predictor of increased severity of an extremity injury. In addition, obesity was found to be an independent predictor of fatal outcomes after motor vehicle crashes. Recent population-based data have shown that obese patients present higher rates of complications than non-obese individuals. Specifically, obese adult trauma patients require more laparotomies and have a significantly higher incidence of postoperative complications, such as respiratory failure, deep vein thrombosis, or multisystem organ failure [149,150].

However, it is important to note that obese patients did, in general, present with older age, lower admission blood pressure, more associated chest injuries, and more severe extremity injuries, so that the differences concerning morbidity and mortality could not solely be linked to obesity itself [147]. Schneider et al. found a negative correlation between BMI and peak GH serum levels in TBI patients. The latter could possibly be explained by an increased vulnerability of somatotrophic cells, putting affected patients at higher risk of developing this disorder in the course of a TBI [29]. This is possibly due to preexisting hypothalamic gliosis processes, given the data in the paragraph above, which are indeed detectable via MRI [125]. Particularly in patients suffering from abdominal obesity with visceral adiposity, a hyperactive and therefore very sensitive hypothalamic-pituitary axis has been documented, which might worsen the outcome after common secondary complications, such as insults, swelling, or hypotension [112,151]. However, it is not clear whether the impairment of GH secretion TBI in adipose patients is due to the injury or simply the result of being overweight. As demonstrated by data from Klose et al. individuals diagnosed with post-traumatic HP presented with adverse lipid profiles and unfavorable body composition (measured by BMI, waist circumference, and body fat mass) 12 months later, but with no significant correlation to insulin-like growth factor 1 (IGF-1) blood levels [56]. Since the latter acts as the main signaling factor in the GH cascade, one could draw the conclusion that additional mechanisms exceeding the GH axis are highly probable.

Thus, as the current evidence is still very fragmentary, simply substituting GH prophylactically to reduce possible complications could become an ineffective measure with no clinical improvement. There is still a need to complete the profiles of individual pathogenic components in obese TBI patients to ensure a solid foundation for personalized treatment plans. For now, the most sensible step is for clinicians to abide by monitoring and controlling the organism’s excessive inflammatory response.

#### 3.2.4. The Metabolic Syndrome

From an endocrinological point of view, obesity is strongly associated with elevated baseline insulin levels, peripheral insulin resistance, and hyperglycemia [112], or, in the worst case, with type II DM. Additionally, blood lipid levels might reach pathologic levels, and it is highly probably that patients will develop hypertension [152]. This clinical picture is called the metabolic syndrome [152] and is associated with many chronic complications not restricted to the cardiovascular system, but also involving other organs, including the brain [134,153]. Given the above considerations, the risks of both metabolic circuits add to each other in this clinical picture. A state of enhanced chronic systemic inflammation compounds itself, generating a vicious cycle that at a certain point is very hard to break. To our knowledge, there are no published data regarding the interactions between TBI and the metabolic syndrome, or the impact of the latter on the post-traumatic outcome. Only one group has shown a prevalence of 50% and a negative correlation between GH responsiveness and BMI in retired professional American football players who experienced multiple head concussions [154]. However, both aspects could not be necessarily linked to pituitary hormonal dysfunction, again stressing the complexity of the interactions between an injured brain and a body suffering this syndrome. The latter and the separation of DM and obesity in highly specialized research groups have surely contributed to the still fragmentary understanding of underlying processes. However, the significantly increased mortality in both patient groups that is combined in patients suffering from metabolic syndrome strongly necessitates joining forces on further research.

### 3.3. Thyroid Dysfunction

In TBI patients, S100β is a very common clinical marker to estimate the presence and degree of damage, indicating both glial cell loss and abnormally increased permeability of the BBB [155,156,157]. Raised serum levels in TBI patients come with more severe radiological findings, higher intracranial pressure, worse GCS scores, and even higher mortality [157,158,159,160,161,162]. Interestingly, the expression of S100β correlates negatively with free thyroxine (T4) levels, the main thyroid hormone [159]. Thus, the relationship between thyroid dysfunction and the potential for structural and functional recovery must be taken into account in the course of TBI. A meta-analysis has revealed a prevalence rate of thyroid dysfunction of about 7% among the adult population in Europe [163]. Since the major percentage covers the number of undiagnosed patients, these data suggest that thyroid dysfunction is a more common disease than one would expect.

#### 3.3.1. Neuroprotective Capacities of Thyroid Hormone in Post-Traumatic CNS

Thyroid hormones are essential for the development, maturation, and functionality of the brain [164]. Recently, the potential role of these hormones in the etiology and manifestation of symptoms following TBI is gaining increasing attention in both clinical and basic research. This is, at least in part, due to the effects of thyroid hormone treatment on neuroprotective capacities observed in experimental animal TBI models. For example, treatment with levothyroxine, a manufactured synthetic form of T4, not only restores hormone levels in serum obtained from rats 24 h after TBI, but does not impact the concentration of the hypothalamic releasing hormone thyroid-stimulating hormone (TSH) or alter expression rates of other important enzymes, which would finally lead to a lower availability of triiodothyronine (T3), the biologically activated form of T4 [165]. Of note, maintenance of T3/T4 serum levels goes hand-in-hand with reduced brain edema and parallels increased transcriptional activation of anti-apoptotic genes, and upregulation of neurotrophic and pleotropic factors and pro-neurogenic factors such as doublecortin (Dcx) and SRY-Box2 (Sox2) [165]. These changes represent an essential step to ensure certain repair mechanisms following damage to the brain parenchyma that include neuroprotection, proper reconstruction of the BBB, and physical fencing of the damaged areas in order to reduce cell death after injury. Along these lines, other groups have also shown in a rat model of acute stroke that post-ischemic thyroid hormone treatment may mediate anti-apoptotic gene expression and reduce reactive gliosis compared to untreated control animals [166].

Taking into account that treatment with T3 resulted in the restoration of hypoxia-inducible factor (HIF) levels in cells cultured under hypoxic conditions in vitro, the promotion of neuronal survival in injured brains upon treatment with thyroid hormone is likely due to the stabilization of oxygen homeostasis via direct or indirect interaction with HIFs [165]. HIFs are also known for their role in cellular adaptation to low oxygen availability during periods of reduced oxygen supply. Moreover, members of the HIF family not only are “master regulators” of oxygen sensing and homeostasis, but also play a crucial role in hypoxia-associated processes, such as vasodilatation, cell migration, signaling, and cell fate specification [167]. It is also conceivable that significantly decreased BBB leakage upon thyroid hormone treatment after brain injury can prevent macrophage infiltration and contribute to pro-survival cell mechanisms, resulting in the suppression of inflammatory responses in the post-traumatic brain parenchyma. Overall, these mechanisms seem to enhance neuroprotective processes, either directly by altering the genome and proteome, or by leading to an improved energy supply in damaged CNS tissue. The latter has been shown to be critical for neurologic outcomes after TBI in humans [63], and is also corroborated by data showing that T3 treatment improved motor and cognitive recovery and reduced lesion size in animals following controlled cortical injury or transient stroke [168,169]. Thus, despite the promising therapeutic potential suggested by these results, further studies on the complex interactions are needed to establish optimal dosages, time frames of application, and combinations of thyroid hormone treatments.

#### 3.3.2. Low Thyroid Hormone Levels Correlate with Bad TBI Outcomes in the Critically Ill

The correlation between abnormally low thyroid hormone levels in critically ill patients and bad prognosis was discovered quite some time ago [158,162]. Usually, during the acute phase of the response to severe physical stress, there is a rapid decline in T3 levels, which is also associated with an increase in concentrations of the biologically inactive reverse T3 (rT3). The severity of the illness can be read out from the rate of T3 decline, whereas mortality rises proportionally. In very fatal cases, a fall of T4 has also been reported, and in milder situations, a drop in T4 levels occurs only when the disease becomes more chronic. This is paralleled with a massive reduction in basal pulsatile TSH secretion activity [80], the hormone released from the hypothalamus controlling thyroid hormone secretion. This, as well as hyperglycemia (please see Section 3.1.3), seems to be caused by cytokines such as TNF-α, IL-1, and IL-6, among other reasons [85].

Low concentrations of free T3 in patients suffering from acute ischemic stroke predict bad outcomes, as expected [170]. Also, high thyroglobulin (Tg) levels have been found to correlate with fatal TBI outcomes [171]. After ruling out other possible pathogenic aspects that could cause this increase, it seems reasonable that damage to the hypothalamic-hypophyseal axis can lead to abnormally excessive release of thyroid hormones and Tg into the bloodstream, with the feedback mechanisms no longer able to maintain physiological balance [171]. In combination with significantly lower TSH concentrations in the acute phase of traumatic brain injury [52], this pathophysiological cascade may underlie the development of post-traumatic hypothyroidism as a consequence of thyroid function abnormalities in TBI patients [159,172].

Thus, at least in experimental animal models and other preclinical data, there is strong evidence that low thyroid hormone levels are central in mediating the worst TBI outcomes. Further work is required to reconcile preclinical and clinical outlooks and allow the establishment of precise treatment protocols for thyroid hormone substitution in TBI patients, hopefully leading to better recovery and outcomes. In terms of screening for early signs of complications related to thyroid function abnormalities, and with the aim of minimizing associated comorbidities, close monitoring of blood levels during the follow-up period seems very beneficial.

## 4. Concluding Remarks

Here, we focus on TBI as a highly variable disorder that not only typically involves structural and functional changes within the brain but culminates in the emergence of reactive cascades affecting the whole organism. Despite growing evidence on the relationship between endocrine dysregulation and pathogenesis in TBI patients, there are still many important aspects that are not fully understood, especially when taking into account that multiple risk factors related to preexisting endocrinopathies are involved in a plethora of injury-induced pathophysiologic mechanisms. Therefore, developing new therapeutic approaches using a combination of drugs to treat the various elements during injury-induced (neuro) endocrine cascades would be of enormous clinical and socioeconomic benefit.

In the meantime, Harris’s original idea of neuroendocrinology could be redefined, since not only a nonfunctioning hypothalamic-hypophyseal axis can affect the whole organism. This means the effects of comorbidities and cognitive or emotional aspects must all be considered to further improve the effectiveness of treatment for TBI patients, opening a field that could be named “endocrinogical neurology”, in which neuroendocrine specialists together with metabolic endocrinologists and neurologists join forces to identify precise and solid diagnostic criteria for TBI patients.
